# Subtle hypometabolism on [^18^F]FDG-PET, yet absent [^123^I]FP-CIT SPECT binding in a patient with spastic paraplegia type 7

**DOI:** 10.1186/s41824-026-00323-6

**Published:** 2026-09-28

**Authors:** Dylan Henssen, Danique L. M. Radder, Anke H. Snijders, Anne I. J. Arens

**Affiliations:** 1 Department of Medical Imaging, Radboud University Medical Center, Nijmegen, The Netherlands; 2 Department of Nuclear Medicine, Universitätsklinikum Leipzig, Leipzig, Germany; 3 Department of Neurology, Radboud University Medical Center, Nijmegen, The Netherlands

**Keywords:** SPG7; Hypometabolism, DaT-scan, [^18^F]FDG, Neuro-imaging, FP-CIT

## Abstract

Nuclear medicine neuroimaging is becoming increasingly important for the diagnosis and differential diagnosis of Parkinson’s disease, atypical parkinsonian syndromes and more rare neurological (movement) disorders. We report on a 46-year-old woman presented with frequent falls, a gradually progressive bipyramidal syndrome with gait ataxia, dysphagia, urinary urgency and -incontinence, low mood with disturbed emotional regulation and a mild asymmetric hypokinetic rigid syndrome. Additionally, she had a blurry vision, asymmetrical ptosis and abnormal vertical saccades. [^18^F]FDG PET/CT of the brain showed unspecific pattern of regional cortical hypometabolism and, more pronounced, striatal hypometabolism. [^123^I]FP-CIT SPECT showed a complete absence of on-target binding. Subsequent genetic testing revealed two variants in *SPG7*. The patient showed a modest favourable response on levodopa. This case illustrates profound neurodegeneration of the presynaptic dopaminergic nigrostriatal neurons in SPG7.

## Interesting image

A 46-year-old woman with a 5 year history of progressive frequency of sudden unexplained falls leading to several fractures was referred for a third opinion. She initially experienced lower limb stiffness, which gradually progressed to a bipyramidal syndrome. There were no complaints regarding mastication, although she reported dysphagia, urinary urgency and incontinence, and a low mood with disturbed emotional regulation. Constipation was absent. The patient furthermore reported episodes of blurred vision, fluctuating in nature, particularly during head movements, accompanied by a sensation that visual input is oscillating or lagging behind. Ophthalmological examination showed no further abnormalities. No hearing impairment was reported. In the anamnesis, a progressive adipositas was mentioned, with a body mass index of 49. Olfactory function was preserved. Speech is described as slightly altered, though not prominently so. No memory impairment was noted. No sensory disturbances were reported. Neurological examination now showed a bipyramidal syndrome with gait ataxia, left sided ptosis, hypometric and abnormal vertical saccades with a vertical gaze palsy and round the house phenomenon and an asymmetrical hypokinetic rigid syndrome (right more than left). There was no weakness in the upper extremities and no evident weakness of the lower extremities was present. Deep tendon reflexes were preserved on the left side, whereas hyperreflexia was observed on the right side. Electromyography showed no evidence of myopathy or anterior horn cell disease. Muscle ultrasound was borderline abnormal (m. flexor digitorum profundus > 2); based on these findings, a neuromuscular disorder could neither be demonstrated nor excluded. In addition, the reference values have not been validated for patients with a high BMI (49.3), which may lead to overestimation of echogenicity. No fasciculations were observed. Electroencephalography demonstrated a background pattern within normal limits, without focal or epileptiform abnormalities during the recording.

**Fig. 1:**
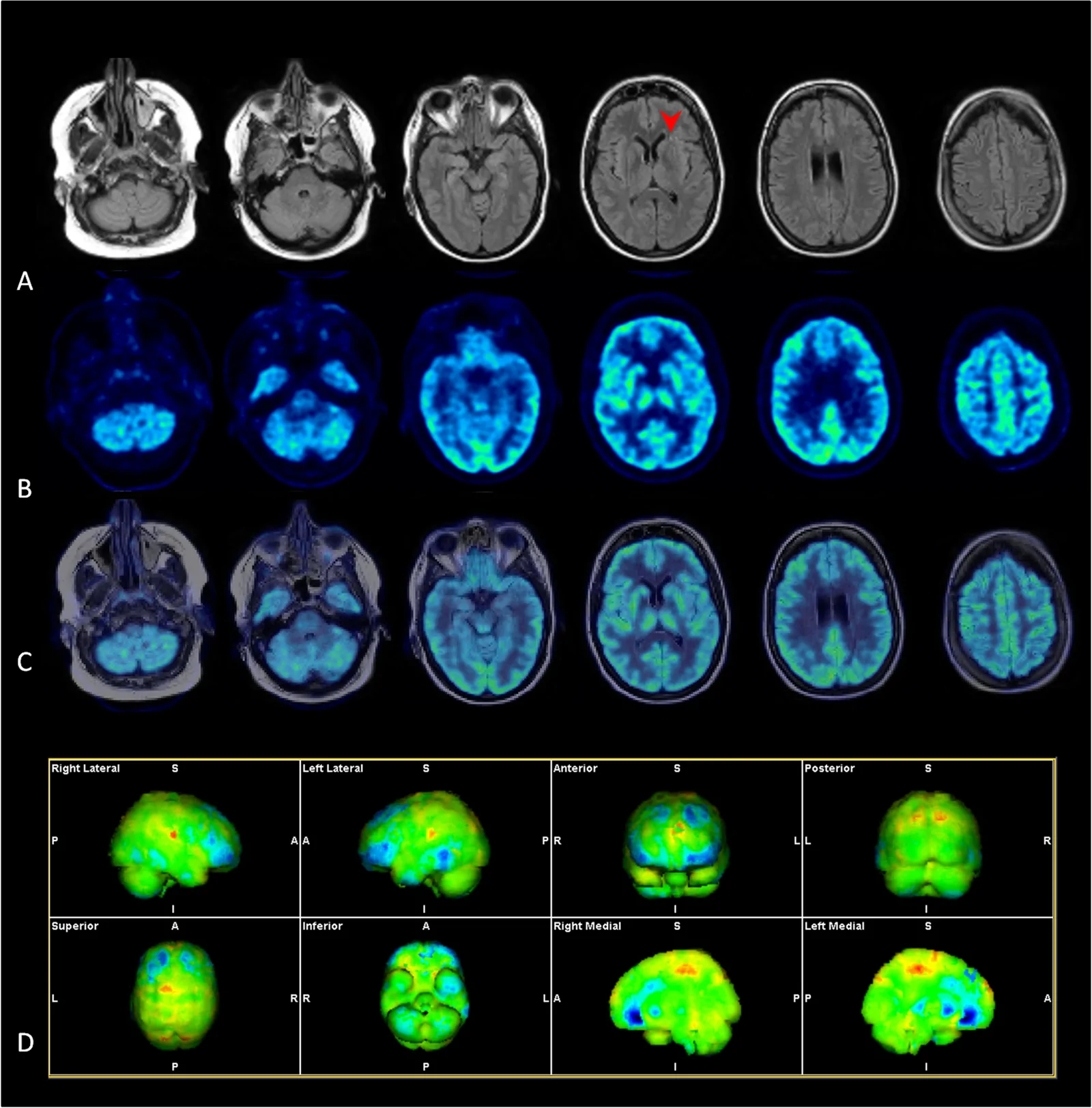
Dedicated [^18^F]FDG PET/CT neuro-imaging and MRI of the patient suffering from Spastic paraplegia type 7. (**A**) Axial slices of fluid attenuated inversion recovery (FLAIR) sequence, revealing a single unspecific white matter hyperintensity in the periventricular white matter in the left frontal lobe (indicated by the red arrowhead). (**B**) Axial slices of the CT-attenuated [^18^F]FDG-PET images at the corresponding levels as depicted in row A. Visual inspection showed no clear patterns of cortical or subcortical hypo- or hypermetabolism. (**C**) Fused axial [^18^F]FDG-PET/FLAIR data showing no abnormal functional-anatomical correlates for any regions with hypo- or hypermetabolism. (**D**) Three-dimensional stereotactic surface projection helped to identify relatively small hypometabolic cortical clusters in the orbitofrontal cortex bilaterally (involving both medial and lateral aspects), the anterior cingulate gyrus bilaterally, extending into the mid portion of the cingulate gyrus, the left superior frontal gyrus and the right inferior and middle temporal gyri. Subcortically, bilateral hypometabolism was observed in the striatum, particularly in the putamen. The insular cortex bilaterally and the infratentorial brain showed normal metabolic activity

In a previous hospital a possible correlation between the adipositas, reduced physical fitness due to deconditioning and frequent falls was hypothesized, as no definite neurodegenerative symptoms could be identified. However, neurological examination was hindered because of the severe adipositas and multiple fractures due to falls. MRI neuroimaging provided no explanation for the clinical symptomatology and subsequent [^18^F]FDG PET/CT imaging of the brain showed no evident pattern of deviant metabolism in cortical and/or subcortical regions. Consequently, no definite diagnosis of a specific neurodegenerative disorder could be made (Fig. 1).

[^18^F]FDG PET imaging of patients with Parkinson’s disease often shows no major abnormality at first glance. On closer inspection and especially on voxel-based statistical analyses, Parkinson’s disease is characterized by a posterior temporoparietal, occipital, and sometimes frontal hypometabolism (especially in patients with cognitive impairment) and relative hypermetabolism of the putamen, pallidum, thalamus, sensorimotor cortex, pons, and cerebellum (Meles et al. 2017). In atypical parkinsonism, the pattern of hypometabolism is dependent on the type parkinsonism (Meyer et al. 2017). Multiple-system atrophy (MSA) patients show regional hypometabolism of the (posterior) putamen, pons, and cerebellum, which may be more pronounced in the striatum or in the pons and cerebellum, depending on the predominant site of degeneration (Meyer et al. 2017). In patients with progressive supranuclear palsy, hypometabolism can be seen in the anterior cingulate gyrus, supplementary motor area, precentral gyrus, and premotor–to–posterior prefrontal cortical areas and insular cortex. Furthermore, regional hypometabolism can be observed in the caudate nucleus, thalamus and midbrain. The cerebellum and sensorimotor cortices can show regional hypermetabolism (Meyer et al. 2017, Martí-Andrés et al. 2020). Corticobasal degeneration, on the other hand, is characterized by a notable asymmetric hypometabolism of the frontoparietal areas, striatum and thalamus where the cortical region of hypometabolism includes the sensorimotor cortex and extends into the cingulate gyrus and premotor–to– posterior prefrontal areas (Meyer et al. 2017).

**Fig. 2:**
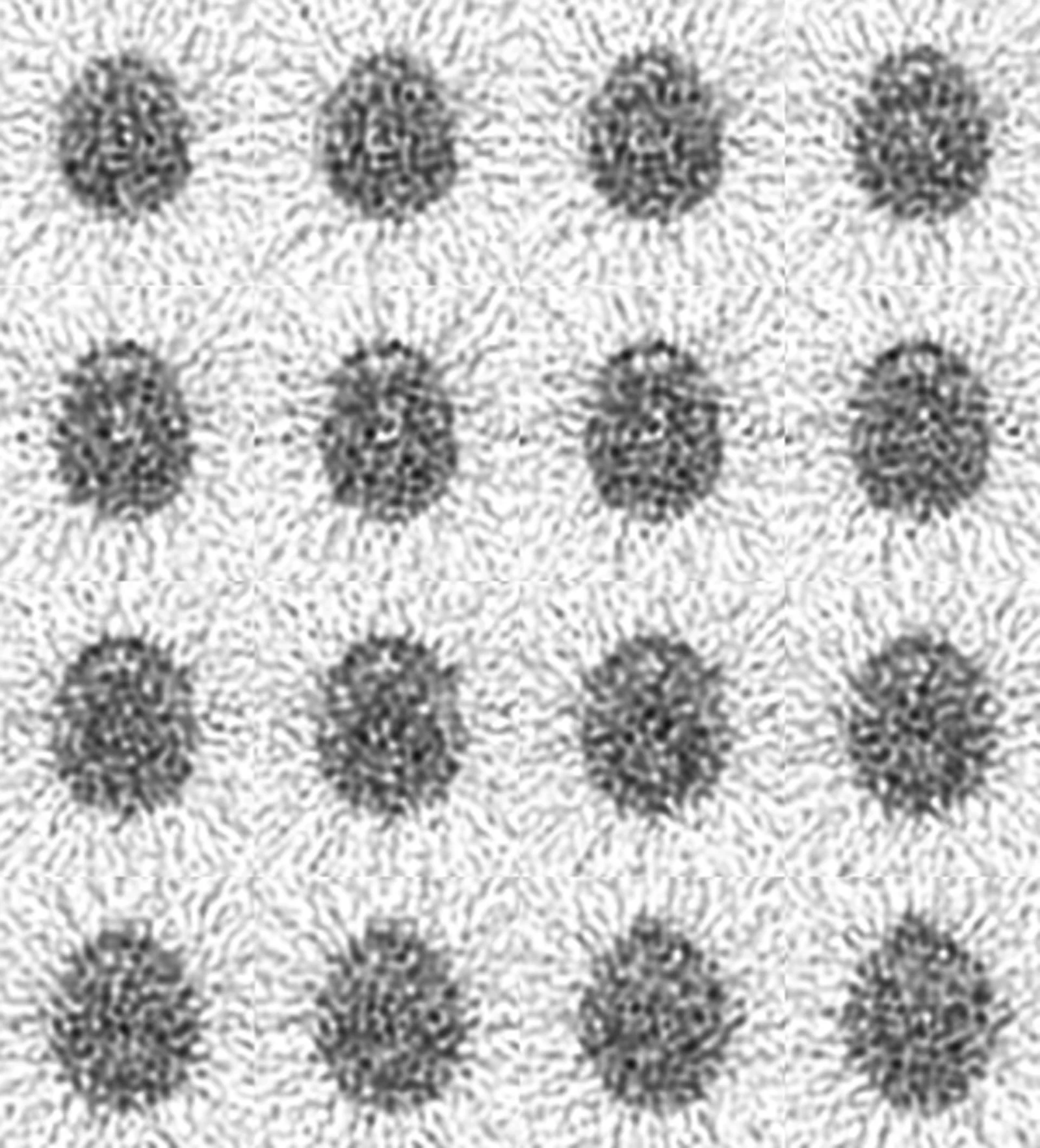
Axial reconstructions of the [^123^I]FP-CIT SPECT investigation in the patient suffering from Spastic paraplegia type 7. No target binding can be observed and a widespread non-specific binding pattern can be observed

Because the initial investigations were considered inconclusive and a neurodegenerative disorder was regarded as unlikely, the patient sought a second opinion at another hospital. No additional investigations were performed, and her obesity-associated physical deconditioning was again considered a possible explanation for the frequent falls. As her symptoms continued to progress, she subsequently presented to our tertiary referral center for a third opinion.After first clinical examinations, adipositas as a sole cause for her discomfort and symptomatology was doubted and an [^123^I]FP-CIT SPECT was performed amongst an EMG, EEG and WES testing a multigene panel of neurodegenerative diseases. The [^123^I]FP-CIT SPECT was completely abnormal, not showing any presynaptic dopamine transporter binding in the striatum bilaterally (Fig. 2). The putamen and the caudate nucleus bilaterally demonstrated no tracer uptake, without asymmetries or a rostrocaudal gradient. She did not take any medication that could interfere with the binding of FP-CIT (she only used enalapril, deltaparine and estradiol). Quality control of the radiopharmaceutical showed no abnormalities. True absence of specific tracer binding is rare and consistent with profound nigrostriatal degeneration.

Further genetic diagnostic testing using WES identified two variants in the *SPG7* gene: one pathogenic and one likely pathogenic. More particularly, heterozygosity was identified for the pathogenic variant c.1045G > A p.(Cys349Ser) located at genomic position 16:89598369 (GRCh37) in the *SPG7* gene (NM_003119.4), which follows autosomal dominant or recessive inheritance. This variant has been reported in patients with autosomal recessive hereditary spastic paraplegia and functional studies have demonstrated a reduction in enzymatic activity (Bonn et al. 2010). Additionally, heterozygosity was detected for the pathogenic variant c.1529 C > T p.(Ala510Val) at genomic position 16:89613145 (GRCh37) in the *SPG7* gene. This variant causes a drastic amino acid substitution and has been described in multiple patients with autosomal recessive spastic paraplegia (Brugman et al. 2008).

Spastic paraplegia type 7 is one of the more common subtypes of autosomal recessive hereditary spastic paraplegias, accounting for 5%-12% of autosomal recessive cases and up to 7% of sporadic adult onset mutations (Brugman et al. 2008). Age at onset is predominantly in the third or fourth decade, although a wide range has been described (Warnecke et al. 2007). Clinical presentation can be either a more pronounced spastic paraplegia phenotype (with or without impaired vibration sense and sphincter disturbances) or a spastic-ataxic complex phenotype (Warnecke et al. 2007). Clinical manifestations can be difficult to distinguish from often concomitant cerebellar ataxia symptoms. Other features that have been described in the spastic-ataxic complex phenotype are: dysphagia, subtle cognitive impairment, optic nerve atrophy, strabismus, supranuclear oculomotor palsy, decreased hearing, scoliosis, amyotrophy and/or sensorimotor neuropathy (Warnecke et al. 2007). Two case-reports described the findings of dopamine transporter SPECT in a patient suffering from spastic paraplegia type 7 (Bellini et al. 2021, Pedroso et al. 2018). Both case reports described the findings of dopamine transporter SPECT using [^99m^Tc]TRODAT-1. In both cases, a bilateral decreased specific binding in the caudate and putamen was observed (Bellini et al. 2021, Pedroso et al. 2018). In one case report, a 55-year old woman presented in this case had a 15-year history of slowly progressive gait disturbance and also had a hypokinetic syndrome, which is a rare clinical finding in SPG7 (Pedroso et al. 2018), whereas in the other case report a 70-year old man with frequent falls is described (Bellini et al. 2021). Together, these cases fit the clinical presentation of our case and it has been reported that SPG7 can mimic MSA-C symptomatology (Bellini et al. 2021). Although revision of the [^18^F]FDG-PET data revealed that the hypometabolism of the putamen could fit the diagnosis of MSA, particularly the MSA-P type (Zhao et al. 2020), the cortical clusters of hypometabolism were still regarded unspecific. Furthermore, the clinical symptomatology did not fit the profile of MSA-C or MSA-P. Furthermore, other Spastic paraplegia types have been described in the literature as clinical mimickers of MSA. In a recent case report by Hesse et al., [^18^F]FDG PET and [^123^I]FP-CIT SPECT was used in a patient with a *SPG4* mutation, showing a diffuse cerebellar hypometabolism, which was not observed in our case. Furthermore, putaminal metabolism was preserved and [^123^I]FP-CIT SPECT showed a severe nigrostriatal dopaminergic nerve cell loss bilaterally, with a higher reduction in the putamen as compared to the caudate nucleus (Hesse et al. 2026). Nevertheless, the complete absence of specific binding as presented in the current case was not described. Therefore, we hypothesize that different *SPG* mutations can result in partially overlapping clinical phenotypes which have more specific molecular imaging biomarkers, however, more research is needed to see whether these sporadic reports can be translated into consistent imaging biomarker findings.

Clinically, our patient was diagnosed with a complex phenotype of SPG7 with parkinsonism. We prescribed levodopa three times daily 31.25 mg, 62.5 mg (after two weeks) and 125 mg (after an additional two weeks), to which the patient showed a modest favorable response.

## Conclusion

In summary, this case illustrates that SPG7 may present with a complex parkinsonian phenotype accompanied by profound presynaptic nigrostriatal dopaminergic degeneration on [123I]FP-CIT SPECT and subtle striatal hypometabolism on [^18^F]FDG PET. Although MRI findings in SPG7 are often non-specific and may include cerebellar or brainstem atrophy, no clear structural correlate explaining the clinical presentation was identified in our patient. The current findings expand the limited literature on molecular neuroimaging in SPG7-associated parkinsonism. However, given the single-case nature of this report, the observed PET and SPECT abnormalities should not be regarded as disease-specific imaging biomarkers at present.

## Data Availability

The data that support the findings of this study are upon reasonable request to the corresponding author at dylan.henssen@radboudumc.nl.

## Abbreviations

[^18^F]FDG: Fluorine-18 fluorodeoxyglucose
[^123^I]FP-CIT: Iodine-123 ioflupane
EEG: Electroencephalography
EMG: Electromyography
MRI: Magnetic Resonance Imaging
PET: Positron Emission Tomography
SPECT: Single Photon Emission Computed Tomography
SPG7: Spastic Paraplegia type 7
WES: Whole Exome Sequencing
